# Psychosoziale Belastung und persistierende Nebenwirkungen bei Langzeitüberlebenden einer Melanomerkrankung im Stadium IV – eine Querschnittsbefragung

**DOI:** 10.1111/ddg.15712_g

**Published:** 2025-07-14

**Authors:** Markus Reitmajer, Norbert Schäffeler, Anne Bach, Lena Nanz, Teresa Amaral, Ulrike Leiter, Lukas Flatz, Andrea Forschner

**Affiliations:** ^1^ Universitäts‐Hautklinik Universitätsklinikum Tübingen; ^2^ Abteilung für Psychosomatische Medizin und Psychotherapie Universitätsklinikum Tübingen

**Keywords:** Langzeitüberlebende, Langzeitüberlebende nach Krebs, Lebensqualität, Melanom im Stadium IV, psychosoziale Belastung, Survivorship, cancer survivors, long‐term survivors, Melanoma stage IV, psychosocial burden, quality of life, survivorship

## Abstract

**Hintergrund:**

Immuncheckpoint‐Inhibitoren und BRAF/MEK‐Inhibitoren haben das Überleben von Patienten mit Stadium‐IV‐Melanomerkrankung verbessert. Die Herausforderungen, mit denen Langzeitüberlebende nach Überwindung der akuten Erkrankungsphase konfrontiert sind, sind jedoch weiterhin unklar. Der Einfluss dieser Therapien auf anhaltende Toxizität und psychosoziale Aspekte wurde nicht umfassend untersucht.

**Material und Methodik:**

Wir führten eine Querschnittsbefragung durch, bei der etablierte Screeninginstrumente wie das Hornheide‐Screening‐Instrument (HSI) und das Distress‐Thermometer (DT) mit der angehängten NCCN (National Comprehensive Cancer Network)‐Problem‐Liste, sowie Melanom‐spezifische Fragen zu persistierenden Nebenwirkungen, sozialen Beeinträchtigungen, emotionalen Bedürfnissen und finanziellen Sorgen verwendet wurden.

**Ergebnisse:**

Insgesamt wurden 159 Patienten mit einer Melanomerkrankung im Stadium IV, die mehr als 5 Jahre nach der Diagnose eines Stadium‐IV‐Melanoms überlebt haben, in diese Studie eingeschlossen. 93 Patienten haben den Fragebogen ausgefüllt. Etwa ein Drittel der DT/HSI‐Werte lag über dem Schwellenwert, was auf den Bedarf an psychoonkologischer Unterstützung hinweist. Mehr als 40% der Patienten mit Stadium‐IV‐Melanom berichteten über persistierende Beschwerden im Zusammenhang mit früheren Behandlungen. Finanzielle Beeinträchtigungen (8%) und Beeinträchtigungen bei der Arbeit (1%) waren unter den befragten Patienten selten.

**Schlussfolgerungen:**

Die hohe Rate an Nebenwirkungen und der hohe psychosoziale Unterstützungsbedarf verdeutlichen die Notwendigkeit von Survivorship‐Programmen in der Nachsorge von Langzeitüberlebenden einer Melanomerkrankung.

## EINLEITUNG

Die neuen Therapieoptionen der Immuncheckpoint‐Inhibition (ICI) und der zielgerichteten Therapie mit BRAF/MEK‐Inhibitoren (TT) haben das Gesamtüberleben (OS) bei Patienten mit Stadium‐IV‐Melanomerkrankung erheblich verbessert.^1^, ^2^, ^3^, ^4^ Bis 2010 überlebten nur 5% der Patienten mit metastasiertem Melanom 5 Jahre.^5^, ^6^, ^7^ Zehn Jahre später war dieser Anteil auf etwa 30% gestiegen,^8^, ^9^, ^10^ und bei Patienten, die als erste Therapielinie eine kombinierte ICI (anti‐PD1/anti‐CTLA4) erhielten, erreichte die Melanom‐spezifische 10‐Jahres‐Überlebensrate unter Studienbedingungen mehr als 50%.^11^, ^12^, ^13^ Während das Gesamtüberleben weiterhin als der wichtigste Maßstab in der Krebsbehandlung gilt, gewinnen psychosoziale Aspekte und Bewältigungsmechanismen mit zunehmender Überlebensdauer der Patienten immer mehr an Bedeutung. Die entscheidende Frage bleibt, welchen Preis Langzeitüberlebende in Bezug auf Toxizität und psychosoziale Belastung zahlen.^14^, ^15^

Es gibt nur begrenztes Wissen über die psychosozialen, physischen und finanziellen Belastungen von Langzeitüberlebenden mit metastasiertem Melanom. Die Daten aus anderen Krebsentitäten lassen jedoch erwarten, dass einige Langzeitüberlebende ohne Einschränkungen leben, während andere weiterhin schwerwiegende Beeinträchtigungen erfahren.^16^, ^17^ In anderen Tumorerkrankungen wurden bereits Survivorship‐Programme etabliert, die heute als unverzichtbar gelten.^18^, ^19^, ^20^, ^21^ Diese Programme adressieren nicht nur die Angst vor Rückfällen, Depressionen und Angststörungen, sondern auch behandlungsspezifische Nebenwirkungen und Toxizität.^22^, ^23^, ^24^, ^25^, ^26^

In einer zuvor publizierten retrospektiven Studie haben wir Therapien, Ansprechraten und tumorspezifische Daten von Melanompatienten charakterisiert, die mehr als 5 Jahre nach dem Fortschreiten in Stadium IV überlebt haben.^8^ Ziel dieser Querschnittsbefragung war es, die psychosozialen Belastungen und Nebenwirkungen dieser Langzeitüberlebenden zu erfassen.

## MATERIAL UND METHODIK

### Studiendesign

Zur Identifikation von Patienten mit einer Stadium‐IV‐Diagnose im Zeitraum vom 01.01.2014 bis 31.12.2017 nutzten wir die institutionelle Datenbank des *Zentralen Malignen Melanom‐Registers* (CMMR). Am 27.11.2023 wurden allen 159 Langzeitüberlebenden papierbasierte Fragebögen zugesandt, und alle bis zum 27.02.2024 eingegangenen Antworten wurden ausgewertet. Eine Erinnerung zur Teilnahme wurde zuvor am 16.01.2024 versandt.

### Einschlusskriterien und Ethikvotum

Die Einschlusskriterien wurden wie folgt definiert: Patienten mit einer Stadium‐IV‐Melanomdiagnose, die nach Eintritt in das Stadium IV mindestens 5 Jahre überlebten und am Stichtag 01.04.2023 noch am Leben waren. Alle Patienten wurden am Universitätsklinikum Tübingen behandelt. Eine schriftliche Einwilligung zur Datendokumentation im Melanomregister für Forschungs‐ und Publikationszwecke lag von allen Teilnehmenden vor. Ein positives Ethikvotum wurde durch die Ethikkommission der Universität Tübingen erteilt (Referenznummer 466/2023BO2). Die Studie wurde gemäß den Empfehlungen der Deklaration von Helsinki durchgeführt.

### Fragebogen

Der Fragebogen umfasste zwei etablierte Screening‐Instrumente: das *Distress Thermometer* (DT) und das *Hornheide‐Screening‐Instrument* (HSI),^15^, ^27^, ^28^, ^29^ sowie selbstentwickelte Melanom‐spezifische Fragen (MSQ), die therapiebedingte Beschwerden wie Nebenwirkungen und Komplikationen von Operationen, Strahlentherapie und systemischen Therapien adressierten. Darüber hinaus wurden die berufliche Situation, Einschränkungen bei Freizeitaktivitäten, finanzielle Sorgen sowie die Inanspruchnahme von psychoonkologischer Unterstützung, Sozialberatung und weiteren Krebspräventionsprogrammen erfasst (siehe Online‐Supplement, Tabelle S1). Zudem enthielt der Fragebogen eine Selbsteinschätzungsfrage (SA) zur Ermittlung, ob die Patienten selbst einen Bedarf an psychoonkologischer Unterstützung wahrnehmen.

Die Werte des DT reichen von 0 (keine Belastung) bis 10 (extreme Belastung), wobei Werte ≥ 5 als über dem Schwellenwert liegend betrachtet werden und auf einen möglichen Bedarf an psychoonkologischer Unterstützung hinweisen.^29^ Dem DT ist die NCCN‐Problemliste beigefügt, die praktische, familiäre, emotionale, spirituelle/religiöse und körperliche Probleme/Belange abdeckt.^28^, ^29^, ^30^ Die Symptomatik „trockener Mund“ wurde hinzugefügt, um die häufig im klinischen Alltag berichtete Sicca‐Symptomatik mit zu erfassen.

Das HSI erfasst den körperlichen Gesundheitszustand, den psychischen Gesundheitszustand, Belastungen/Beschwerden, die unabhängig von der Melanomerkrankung bestehen, die Verfügbarkeit einer Vertrauensperson, die Belastung eines Familienmitglieds durch die Melanomerkrankung, innere Unruhe sowie den Informationsstand hinsichtlich der Erkrankung und Behandlung. Werte ≥ 4 Punkte deuten auf einen Bedarf an psychosozialer Unterstützung hin.^15^, ^31^

### Statistische Analyse

Die demografischen und klinischen Daten wurden deskriptiv analysiert. Korrelationen zwischen den im HSI, DT und der Selbsteinschätzungsfrage (SA) erfassten Werten wurden mittels Spearman‐Rangkorrelation (zweiseitig) berechnet. Eine binäre logistische Regression für jede NCCN‐Problematik sowie die im MSQ erfassten Daten wurde durchgeführt, um potenzielle Faktoren zu identifizieren, die signifikant mit einer erhöhten Belastung im DT oder dem Bedarf an psychoonkologischer Unterstützung (Cut‐off ≥ 5) assoziiert sind. Die Analysen wurden mit der Software IBM^®^ SPSS^®^ Statistics 28 (IBM, Armonk, USA) durchgeführt. Unvollständige Fragebögen wurden unter Verwendung der verfügbaren Daten einbezogen. Grafiken wurden mit GraphPad PRISM^®^ 9.5.0 (Dotmatics, Boston, USA) erstellt.

## ERGEBNISSE

### Charakterisierung der Patientenkohorte

Insgesamt haben 93 von 159 Patienten den Fragebogen ausgefüllt: 38 Frauen (40,9%) und 55 Männer (59,1%). Das mediane Alter beim Eintritt in Stadium IV lag bei 63 Jahren (Bereich: 25–83 Jahre). Die mediane Zeitspanne zwischen dem Eintritt in Stadium IV und dem letzten Kontakt betrug 6 Jahre (Bereich: 5–9 Jahre) (Tabelle 1). Neun Fragebögen (5,7%) konnten aufgrund unbekannter Adressänderungen nicht zugestellt werden. Eine Übersicht über die Merkmale aller kontaktierten Patienten ist in Tabelle 1 ersichtlich.

**TABELLE 1:** Patientencharakteristika.

Charakterisierung der Patientenkohorte	Patienten, die den Fragebogen beantwortet haben		Patienten, die nicht auf den Fragebogen geantwortet haben	
	*n*	*Prozent [Bereich]*	*n*	*Prozent [Bereich]*
Anzahl an Patienten	93	100	66	100
Geschlecht				
Weiblich	38	40,9	32	48,5
Männlich	55	59,1	34	51,5
Jahre seit der Diagnose zum Zeitpunkt der Befragung, Median [Bereich]	11	[6–36]	10	[6–33]
Alter zum Zeitpunkt der Stadium‐IV‐Diagnose, Median [Bereich]	63	[25–83]	59	[30–85]
Jahre zwischen dem Eintritt in Stadium IV und dem letzten Kontakt, Median [Bereich]	6	[5–9]	6	[5–8]
Melanom‐Typ				
Haut	71	76,3	51	77,3
Okkult	9	9,7	8	12,1
Akral‐lentiginös	6	6,5	3	4,5
Uveal	5	5,4	3	4,5
Schleimhaut	2	2,2	1	1,5
Fernmetastasen				
Lunge	50	53,8	39	59,1
Leber	31	33,3	18	27,3
Zentrales Nervensystem	22	23,7	15	22,3
BRAF^V600^‐Mutation				
Wildtyp	48	51,6	32	48,5
Mutation vorhanden	39	41,9	27	40,9
Unbekannt	6	6,5	7	10,6
Erhielt zum Zeitpunkt der Befragung (01.04.2023) noch eine systemische Therapie	14	15,1	7	10,6
ICI zu irgendeinem Zeitpunkt				
Ja	72	77,4	42	63,6
Nein	21	22,6	24	36,4
TT zu irgendeinem Zeitpunkt				
Ja	22	23,7	14	21,2
Nein	71	76,3	52	78,8
Nichtadjuvante Systemtherapielinien				
Eine	80	86,0	51	77,3
Zwei	36	38,7	28	42,4
Drei oder mehr	25	26,9	19	28,8
Ohne jegliche nichtadjuvante Systemtherapie (28 Patienten)	13	14,0	15	22,7
Nur adjuvante Systemtherapie	6	6,5	5	7,6
Nur adjuvanten Strahlentherapie	2	2,2	2	3,0
Nur OP/Stereotaxie/ Radiofrequenzablation	5	5,4	8	12,1
Strahlentherapie zu irgendeinem Zeitpunkt				
Ja	39	41,9	23	34.8
Nein	54	58,1	43	65.2
Strahlentherapie				
Gehirn	12	12,9	9	13.6
Weichgewebe/regionale Lymphknoten	21	22,6	11	16.7

### Korrelation, der im HSI und DT erfassten Belastungswerten

Wir führten eine zweiseitige Spearman‐Korrelationsanalyse durch, um den möglichen Zusammenhang zwischen HSI, DT und der Selbsteinschätzungsfrage (SA) zu untersuchen. Ein starker Zusammenhang wurde zwischen HSI und DT (ρ = 0,674, n = 87) festgestellt, während moderate Korrelationen zwischen HSI/SA (ρ = 0,457, n = 90) und DT/SA (ρ = 0,351, n = 87) beobachtet wurden. Sowohl HSI (n = 29/90, 32,2%) als auch DT (n = 32/88, 36,4%) zeigten einen ähnlichen Prozentsatz von Patienten mit Bedarf an psychoonkologischer Unterstützung an. Bemerkenswert ist, dass der Prozentsatz der Patienten, die in der SA einen Bedarf an psychoonkologischer Unterstützung angaben (n = 11/90, 12,2%), niedriger war als der aus den professionellen Bewertungsinstrumenten (HSI, DT) abgeleitete Wert (siehe Online‐Supplement, Tabelle S2). Mehr als ein Drittel der Langzeitüberlebenden mit Melanom berichteten über DT‐Werte oberhalb des Schwellenwerts (36,4%, n = 32/88).

### Körperliche Beschwerden ist die dominierende NCCN‐Problemlistenkategorie

**ABBILDUNG 1:**
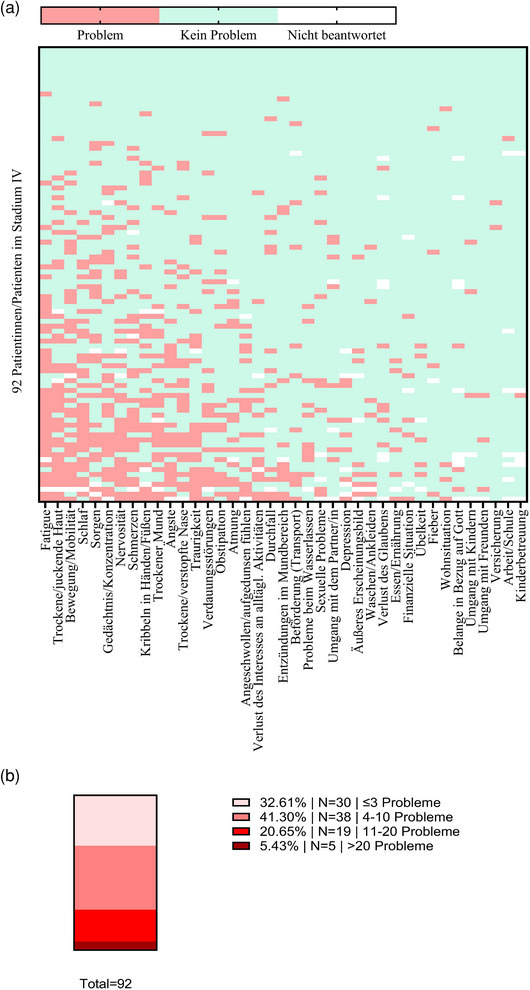
Ergebnisse der NCCN‐Problemliste. (a) Die *Heatmap* bietet einen visuellen Überblick über alle genannten Probleme der 92 Stadium‐IV‐Patienten, die den NCCN‐Fragebogen ausgefüllt haben. Horizontale Achse: Die häufigsten Probleme sind auf der linken Seite der *Heatmap* angeordnet. Vertikale Achse: Patienten, die die meisten Problemen erfahren, befinden sich am unteren Rand der *Heatmap*. (b) Die Patienten wurden basierend auf der NCCN‐Problemliste in vier Gruppen unterteilt: Gruppe 1 berichtete von ≤ 3 Problemen, Gruppe 2 berichtete von 4–10 Problemen, Gruppe 3 berichtete von 11–19 Problemen und Gruppe 4 berichtete von ≥ 20 Problemen.

Zweiundneunzig der 93 Patienten mit Stadium‐IV‐Melanom füllten die NCCN‐Problemliste zumindest teilweise aus. Dabei war Müdigkeit/Fatigue das häufigste genannte Problem (43,3%, n = 39/90) (Abbildung 1a). Körperliche Beschwerden wurden am häufigsten genannt, während „familiäre“, „praktische“ und „spirituelle/religiöse“ Belange nur selten erwähnt wurden (Tabelle 2). In Bezug auf die Gesamtzahl der genannten Probleme berichteten etwa ein Drittel der Patienten (32,6%, n = 30) von insgesamt ≤ 3 Problemen, 41,3% (n = 38) gaben 4–10 Probleme an, und 20,7% (n = 19) berichteten von ≥ 11 Problemen. Eine Untergruppe von 5,4% (n = 5) gab mehr als 20 Probleme an (Abbildung 1b). Etwa ein Fünftel der Patienten hatte mindestens einmal Kontakt zur psychoonkologischen Beratung oder Sozialberatung (Tabelle 3).

**TABELLE 2:** NCCN‐Problem‐Liste, geordnet von der häufigsten zur seltensten Angabe.

NNCN‐Problemliste	Melanom AJCC Stadium IV		Problemkategorie
	*Positiv/total*	*Prozent*	
Fatigue/Müdigkeit	39/90	43,3	Körperlich
Trockene/juckende Haut	35/89	39,3	Körperlich
Bewegung/Mobilität	32/90	35,6	Körperlich
Schlaf	32/91	35,2	Körperlich
Sorgen	29/89	32,6	Emotional
Gedächtnis/Konzentration	29/90	32,2	Körperlich
Nervosität	29/90	32,2	Emotional
Schmerzen	28/89	31,5	Körperlich
Kribbeln in Händen/Füßen	24/89	27,0	Körperlich
Trockener Mund	24/89	27,0	Körperlich
Ängste	23/91	25,3	Emotional
Trockene/verstopfte Nase	23/91	25,3	Körperlich
Traurigkeit	22/91	24,2	Emotional
Verdauungsstörungen	19/91	20,9	Körperlich
Obstipation	17/92	18,5	Körperlich
Atmung	15/90	16,7	Körperlich
Angeschwollen/aufgedunsen fühlen	15/90	16,7	Körperlich
Verlust des Interesses an alltäglichen Aktivitäten	13/90	14,4	Emotional
Durchfall	13/90	14,4	Körperlich
Entzündungen im Mundbereich	11/91	12,1	Körperlich
Beförderung/Transport	10/91	11,0	Praktisch
Probleme beim Wasserlassen	10/91	11,0	Körperlich
Sexuelle Probleme	9/89	10,1	Körperlich
Umgang mit dem Partner/in	8/89	9,0	Familiär
Depression	7/87	8,0	Emotional
Äußeres Erscheinungsbild	7/88	8,0	Körperlich
Waschen/Ankleiden	7/91	7,7	Körperlich
Verlust des Glaubens	6/85	7,1	Spirituell/Religiös
Essen/Ernährung	6/91	6,6	Körperlich
Finanzielle Situation	5/89	5,6	Praktisch
Übelkeit	5/90	5,6	Körperlich
Fieber	4/91	4,4	Körperlich
Wohnsituation	4/92	4,3	Praktisch
Belange in Bezug auf Gott	3/83	3,6	Spirituell/Religiös
Umgang mit Kindern	3/90	3,3	Familiär
Umgang mit Freunden	3/91	3,3	Familiär
Versicherung	2/92	2,2	Praktisch
Arbeit/Schule	1/85	1,2	Praktisch
Kinderbetreuung	1/86	1,2	Praktisch

Jeder Problemkategorie wurde eine Farbe zugeordnet: praktische Belange = gelb, emotionale Probleme = orange, familiäre Probleme = grün, spirituell/religiöse Probleme = blau, körperliche Probleme = rot.

**TABELLE 3:** Bewertung der Melanom‐spezifischen Fragen (MSQ) im Fragebogen.

Melanom‐spezifische Fragen	Positiv/total	Prozent	Median der Jahre seit der letzten Therapie [Bereich]
*Leidet immer noch unter Problemen aufgrund einer medikamentösen Systemtherapie*			
Ja	37/80	46,3	6 [0–11]
Nein	43/80	53,8	6 [0–9]
Nicht beantwortet	0/80	0	
*Leidet immer noch unter Problemen aufgrund von operativen Verfahren*			
Ja	39/93	41,9	11 [7–29]
Nein	50/93	53,8	11 [7–37]
Nicht beantwortet	4/93	4,3	12 [11–16]
*Leidet immer noch unter Problemen aufgrund einer Strahlentherapie*			
Ja	17/39	43,6	8 [2–13]
Nein	22/39	56,4	8 [3–14]
Nicht beantwortet	0/39	0	
*Erlebt finanzielle Beeinträchtigungen aufgrund der Melanomerkrankung Stadium IV*			
Ja	7/93	7,5	
Nein	81/93	87,1	
Nicht beantwortet	5/93	5,4	
*Erlebt Einschränkungen in der Freizeit aufgrund der Melanomerkrankung Stadium IV*			
Ja	30/93	32,3	
Nein	57/93	61,3	
Nicht beantwortet	6/93	6,5	
*Beeinträchtigung durch die Melanomerkrankung am Arbeitsplatz*			
Ja	1/93	1,1	
Nein	29/93	31,2	
Arbeitet nicht mehr/pensioniert/berentet	60/93	64,5	
Nicht beantwortet	3/93	3,2	
*Unterstützung durch die psychoonkologische Beratung*			
Ja	21/93	22,6	
Nein	61/93	65,6	
Nicht beantwortet	11/93	11,8	
*Unterstützung durch die Sozialberatung*			
Ja	19/93	20,4	
Nein	65/93	70,0	
Nicht beantwortet	9/93	9,7	
*Nimmt regelmäßig an Vorsorgeprogrammen anderer Krebsentitäten teil (Darm‐, Brust‐, Prostatakrebs)*			
Ja	73/93	78,5	
Nein	17/93	18,3	
Nicht beantwortet	3/93	3,2	
*Fühlt sich ausreichend über die Risiken und Chancen der medikamentösen Systemtherapie bei Melanom‐Erkrankung informiert*			
Ja	76/93	81,7	
Nein	15/93	16,1	
Nicht beantwortet	2/93	2,2	

### Finanzielle und berufliche Beeinträchtigungen durch die Melanomerkrankung im Stadium IV

Etwa 8% (n = 7/93) der Patienten gaben an, dass finanzielle Beeinträchtigungen durch die Melanomerkrankung im Stadium IV verursacht wurden. Nur ein Patient berichtete von Einschränkungen am Arbeitsplatz, während die Mehrheit der Patienten (64,5%, n = 60/93) nicht mehr berufstätig war. Etwa ein Drittel der Patienten (31,2%, n = 29/93) fühlte sich am Arbeitsplatz nicht durch die Melanom‐Diagnose eingeschränkt. Es ist anzumerken, dass die Umfrage nicht erfasste, ob die Patienten ihre Arbeit aufgrund des Melanoms aufgegeben haben. Etwa ein Drittel (32,3%, n = 30/93) gab an, in ihrer Freizeit durch die Melanom‐Erkrankung eingeschränkt zu sein (Tabelle 3). Der häufigste Grund hierfür war eine Einschränkung der Bewegung/Mobilität (n = 10). Weder finanzielle noch berufliche Einschränkungen waren signifikant mit DT‐Werten ≥ 5 assoziiert.

### Mehr als 40% der Patienten mit Melanomerkrankung im Stadium IV berichten von anhaltenden Beschwerden aufgrund früherer Behandlungen

**ABBILDUNG 2:**
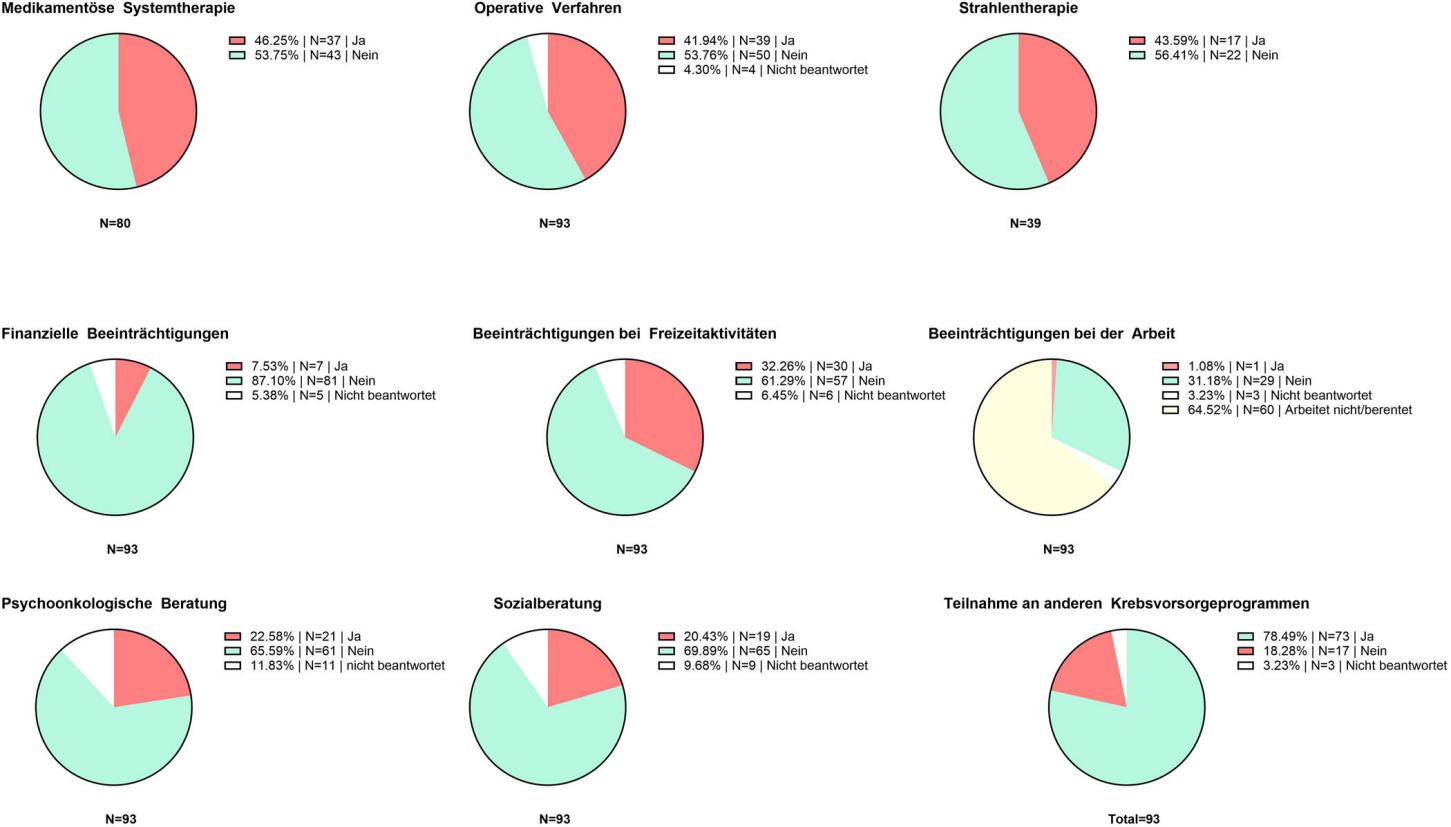
Ergebnisse der Melanom‐spezifischen Fragen (MSQ). Die Abbildung zeigt Nebenwirkungen und Probleme im Zusammenhang mit medikamentöser Systemtherapie, chirurgischen Interventionen und Strahlentherapie sowie Auswirkungen auf Freizeit, Finanzen und Arbeit.

Sechsundvierzig Prozent (n = 37/80) der Patienten, die mindestens eine medikamentöse Systemtherapie erhalten haben, berichteten von anhaltenden körperlichen Beschwerden, die sie auf frühere Systemtherapien zurückführen (Abbildung 2, Tabelle 3). Am häufigsten genannt wurden Hautprobleme (n = 23), trockener Mund (n = 15) und Gelenkbeschwerden (n = 15). Die mediane Zeit seit dem Ende der letzten Therapie betrug 6 Jahre, mit einer Spannweite von 0 bis 11 Jahren. Dreiunddreißig dieser Patienten (89,2%) hatten mindestens einen ICI‐Zyklus erhalten, und neun (18,9%) hatten mindestens eine Therapielinie mit TT erhalten. Eine ebenso hohe Zahl (43,6%, n = 17/39) der Patienten berichteten von anhaltenden Symptomen aufgrund einer früheren Strahlentherapie (Abbildung 2, Tabelle 3). Am häufigsten genannt wurden Bewegungseinschränkungen (n = 7), Ödeme/Schwellungen (n = 5) und Taubheitsgefühl der Haut (n = 5). Die mediane Zeit seit der letzten Strahlentherapie betrug 8 Jahre, mit einer Spannweite von 2 bis 23 Jahren. Von den Langzeitüberlebenden im Stadium IV leiden 41,9% (n = 39/93) weiterhin unter körperlichen Beschwerden, die sie auf frühere chirurgische Eingriffe zurückzuführen sind (Abbildung 2, Tabelle 3). Von den Patienten, die eine zerebrale Strahlentherapie erhalten hatten, berichteten 41,8% (n = 5/12) von Gedächtnisproblemen und Konzentrationsstörungen. Die mediane Zeit seit dem letzten chirurgischen Eingriff betrug 11 Jahre, mit einer Spannweite von 7 bis 29 Jahren. Derzeit berichten 22 Patienten von Taubheitsgefühl der Haut, 17 von Bewegungseinschränkungen und 15 von Ödemen aufgrund früherer chirurgischer Eingriffe. Allerdings gaben 81,7% (n = 76/93) der Patienten an, dass sie sich gut über die potenziellen Risiken und Nutzen der angewandten systemischen Therapien informiert wurden. Ein Großteil der Patienten (78,5%, n = 73/93) gab an, regelmäßig an weiteren Krebsvorsorgeuntersuchungen, wie zum Beispiel an Programmen zur Prävention von Darm‐, Brust‐ oder Prostatakrebs, teilzunehmen (Tabelle 3).

### Faktoren, die signifikant mit DT‐Werten ≥ 5 assoziiert sind

Es wurde eine binäre logistische Regression für jedes Problem der NCCN‐Problemliste sowie für die Probleme des MSQ durchgeführt. Dabei wurden neun Faktoren identifiziert, die signifikant mit DT‐Werten ≥ 5 assoziiert sind, was auf einen Bedarf an psychoonkologischer Unterstützung hinweist (Tabelle 4). Sechs dieser signifikanten Faktoren stammen aus der NCCN‐Problemliste: Beförderung/Transport, Ängste, Nervosität, Schlaf, Bewegungseinschränkungen und trockene/juckende Haut. Zwei Faktoren aus dem MSQ waren ebenfalls signifikant: anhaltende Probleme durch medikamentöse Systemtherapie und Einschränkungen der Freizeitaktivitäten aufgrund des Melanoms. Darüber hinaus war die positive Beantwortung der Frage zur SA, ob psychoonkologische Hilfe benötigt wird, signifikant mit einer Überschreitung des DT‐Schwellenwerts assoziiert. Nicht signifikante Faktoren sind in der Tabelle S3 (siehe Online‐Supplement) aufgelistet.

**TABELLE 4:** Signifikante Faktoren, die mit einer erhöhten Belastung im DT (Cut‐off ≥ 5) assoziiert sind.

Faktoren	Kategorie	n	B	Standardfehler	p	Odds ratio [Exp(B)]	95%‐KI Exp(B)		Cox‐/Snell R^2^	Nagelkerkes R^2^
							*Untere Grenze*	*Obere Grenze*		
Bewegung/Mobilität	NCCN	73	5,262	2,097	0,012	192,953	3,167	11754,628	0,522	0,733
Schlaf	NCCN	73	4,283	1,691	0,011	72,485	2,636	1993,261	0,522	0,733
Trockene/juckende Haut	NCCN	73	3,645	1,526	0,017	38,281	1,922	762,451	0,522	0,733
Ängste	NCCN	83	2,393	0,870	0,006	10,943	1,989	60,216	0,368	0,507
Beförderung/Transport	NCCN	78	2,381	1,141	0,037	10,812	1,156	101,137	0,168	0,233
Selbsteinschätzung bezüglich des Bedarfs an psychoonkologischer Unterstützung	MSQ	87	1,754	0,721	0,015	5,778	1,407	23,720	0,075	0,102
Nervosität	NCCN	83	1,558	0,688	0,024	4,749	1,232	18,302	0,368	0,507
Weiterhin beeinträchtigt/Neben‐wirkungen durch die medikamentöse Systemtherapie	MSQ	80	1,407	0,521	0,007	4,082	1,469	11,343	0,142	0,193
Beeinträchtigung durch die Melanomerkrankung in Freizeitaktivitäten	MSQ	83	1,218	0,489	0,013	3,379	1,295	8,816	0,074	0,102

*Abk*.: NCCN, National Comprehensive Cancer Network Problem‐Liste; MSQ, Melanom‐spezifische Fragen; B, Regressionskoeffizient B; Exp(B), Exponentialfunktion von B; KI, Konfidenzintervall

## DISKUSSION

Langzeitüberlebende von Krebs werden oft als geheilt betrachtet; jedoch bleibt es unklar, ob sie im Vergleich zur Allgemeinbevölkerung wirklich als vollständig gesund und resilient gelten. Bei anderen Krebsentitäten wurden persistierende therapiebedingte Nebenwirkungen sowie psychosoziale Beeinträchtigungen bei Langzeitüberlebenden bereits nachgewiesen.^21^, ^32^, ^33^, ^34^, ^35^, ^36^ Soweit uns bekannt ist, handelt es sich hierbei um die erste Querschnittsbefragung unter langzeitüberlebenden Melanom‐Patienten, die mindestens 5 Jahre nach Eintritt in das Stadium IV überlebt haben. Der Fokus der Umfrage liegt auf der Erfassung der psychoonkologischen Situation, der finanziellen Belastungen durch die Erkrankung sowie der potenziellen langfristigen Nebenwirkungen. Hierbei kommen etablierte Screening‐Instrumente (DT, HSI) sowie individuell auf die Melanomerkrankung angepasste Fragen zum Einsatz.

In einer kürzlich durchgeführten Studie zu langzeitüberlebenden Melanompatienten, die mit ICI bei inoperablem Melanom in den Stadien III und IV behandelt wurden und mehr als 12 Monate überlebt haben, berichteten die Patienten am häufigsten von Symptomen wie Müdigkeit (28%), Gelenkschmerzen (17%) und Muskelschmerzen (12%).^37^ Vergleichbar mit diesen Ergebnissen zeigte sich in unserer Kohorte, dass Müdigkeit/Fatigue (43%) und Bewegungs‐ beziehungsweise Mobilitätsprobleme (36%) zu den häufigsten Beschwerden zählen. Juckende oder trockene Haut (39%) wurde als zweithäufigstes Problem in der NCCN‐Problemliste identifiziert und konnte außerdem als signifikanter Faktor im Zusammenhang mit DT‐Werten ≥ 5 assoziiert werden. Patienten gaben ebenso an, dass die Haut infolge einer früheren systemischen medikamentösen Therapie das am häufigsten betroffene Organ ist. Ein möglicher Grund hierfür könnte sein, dass kutane immunvermittelte Nebenwirkungen (immune‐related adverse events, irAE) mit einem verbesserten Therapieansprechen unter ICI in Zusammenhang stehen und daher in dieser Kohorte von Langzeitüberlebenden häufiger auftreten. Es gibt mehrere Hinweise in der Literatur, die darauf hinweisen, dass das Auftreten kutaner irAE (insbesondere Vitiligo) mit einem verbesserten Therapieansprechen unter ICI assoziiert sein könnte.^38^, ^39^, ^40^ Während nur ein kleiner Teil der Patienten (15%) zum Zeitpunkt der Befragung noch eine systemische Therapie erhält, ist davon auszugehen, dass diese irAE bei den meisten Betroffenen auch nach Beendigung der Behandlung weiterhin bestehen.

In unserer Befragung gaben über 40% der Patienten mit einem Stadium‐IV‐Melanom an, weiterhin unter Problemen aufgrund früherer Behandlungen zu leiden. Dies entspricht den Ergebnissen einer früheren Studie, die eine Rate von 43% chronischer irAE bei Patienten mit Stadium‐III–IV‐Melanom unter Anti‐PD‐1‐Therapie zeigte.^41^ Wir beobachteten, dass diese anhaltenden Nebenwirkungen signifikant mit psychoonkologischen Belastungswerten ≥ 5 im DT assoziiert werden konnten. Fast 90% aller Patienten erhielten mindestens einen Zyklus einer ICI‐Therapie, während nur etwa 20% mindestens einen Zyklus einer TT‐Therapie durchliefen. Dies könnte mit Beobachtungen im adjuvanten Setting übereinstimmen, die zeigen, dass TT im Vergleich zu ICI eine höhere, jedoch vorübergehende, und eine geringere anhaltende Toxizität verursacht. In dieser retrospektiven Analyse wurden anhaltende irAE bei 3% (95%‐Konfidenzintervall [KI] 1,6–5,3%) der TT‐Gruppe und bei 26,3% (95%‐KI 20,5–32,9%) der ICI‐Gruppe festgestellt.^42^ Bemerkenswert ist, dass wir keine signifikante Assoziation zwischen DT‐Werten ≥ 5 und anhaltenden Nebenwirkungen nach Strahlentherapie oder chirurgischen Eingriffen feststellen konnten. Aufgrund der kleinen Stichprobengröße (n = 80) und breiter Konfidenzintervalle in einigen Untergruppen könnte es unserem statistischen Modell an Präzision fehlen. Größere multizentrische Studien sind hier erforderlich, um endgültige Schlussfolgerungen ziehen zu können.

Während die sozioökonomischen Folgen bei Langzeitüberlebenden anderer Krebserkrankungen, wie Arbeitslosigkeit, Frühverrentung und daraus resultierende finanzielle Belastungen, gut dokumentiert sind, bleibt die Relevanz dieses Themas bei Langzeitüberlebenden mit Stadium‐IV‐Melanom unbeantwortet.^32^, ^43^ In der vorliegenden Studie konnten wir zeigen, dass kein Problem aus der Kategorie der praktischen Sorgen unter den zehn häufigsten angegebenen Beschwerden rangierte. Nur 8% der Patienten berichteten von finanziellen Einschränkungen. Hierbei zu erwähnen ist, dass diese Ergebnisse ausschließlich Hautkrebszentren innerhalb der DeCOG betreffen, in denen rechtlich geregelte soziale Schutzmechanismen in Bezug auf Finanzen und Krankenversicherung gut etabliert sind. Daher könnten Probleme der praktischen und familiären Problemkategorie in Ländern mit anderen Gesundheits‐ und Sozialversicherungssystemen von höherer Relevanz sein. Zudem lag das Eintrittsalter in das Stadium‐IV‐Melanom in der vorliegenden Kohorte nahe dem normalen Renteneintrittsalter in Deutschland. Dies spiegelt sich auch in dem sehr geringen Anteil (1%) der Patienten wider, die angaben, aufgrund ihres Melanoms bei der Arbeit eingeschränkt zu sein. Von unseren Patienten sind 67% nicht mehr berufstätig. Allerdings wurde nicht erfasst, ob diese Patienten aufgrund des regulären Rentenalters oder aufgrund des Melanoms oder anderer Gründe vorzeitig in Rente gegangen sind.

Bei einem Vergleich der erfassten Distress‐Thermometer‐Werte unserer Langzeitüberlebenden im Stadium IV mit einer bereits publizierten Kohorte von Stadium‐I‐Patienten (nicht ausschließlich Langzeitüberlebende) zeigte sich, dass die vorliegende Studienkohorte der Langzeitüberlebenden im Stadium IV einen geringeren Prozentsatz an Überschreitungen des Schwellenwerts aufweist.^44^ Ursächlich könnten erfolgreiche Anpassungs‐ und Krankheitsbewältigungsprozesse unter Langzeitüberlebenden sein. Andererseits könnten auch effektive Bewältigungsstrategien zur Langzeitüberlebensrate im Stadium IV beigetragen haben.^45^ Nahezu 80% der befragten Patienten gaben zudem an, regelmäßig an Krebsfrüherkennungsprogrammen anderer Krebsarten teilzunehmen, was auf eine Form von bewusstem Gesundheitsverhalten hindeuten könnte.

In der vorliegenden Studie wurden hohe Raten an psychosozialer Belastung und anhaltenden Nebenwirkungen erfasst. Die befragte Studienkohorte besteht aus Langzeitüberlebenden einer Melanomerkrankung, die mindestens 5 Jahre nach dem Eintritt in das Stadium IV überlebt haben. Ein direkter Vergleich mit Personen, die nicht an einem Melanom erkrankt sind, wurde jedoch in dieser Querschnittsbefragung nicht durchgeführt. Daher können wir nicht abschließend bestätigen, dass die von den Patienten berichteten Probleme ausschließlich durch das Melanom und nicht durch andere potenzielle Einflussfaktoren verursacht wurden.

Unsere Daten legen nahe, dass Überlebende einer Melanomerkrankung im Stadium IV potenziell anders betrachtet werden sollten als Langzeitüberlebende anderer Krebsarten. Finanzielle Probleme und soziale Beeinträchtigungen scheinen von untergeordneter Bedeutung zu sein, während körperliche Beschwerden häufig auftreten. Hohe Raten an anhaltenden Nebenwirkungen wurden identifiziert, was die Bedeutung der Implementierung von Krebsüberlebensprogrammen in der Nachsorge von Melanom‐Patienten unterstreicht. Weitere multizentrische Studien weltweit sind erforderlich, um diese heterogene und wachsende Patientengruppe vollständig zu verstehen.

## DANKSAGUNG

Open access Veröffentlichung ermöglicht und organisiert durch Projekt DEAL.

## INTERESSENKONFLIKT

M.R. erhielt eine Förderung im Rahmen des Junior Clinician Scientists Programms der Universität Tübingen (Antragsnummer 523‐0‐0) sowie Reisekostenzuschüsse von Almirall Hermal und Pierre Fabre, außerhalb der eingereichten Arbeit. T.A. berichtet über persönliche Honorare von BMS, CeCaVa, Novartis, Philogen, Pierre Fabre und Delcath sowie über institutionelle finanzielle Unterstützung von iFIT, Neracare, Novartis, Sanofi und SkylineDX. Zudem erhielt sie ein institutionelles Forschungsstipendium von Novartis. Sie ist Vorsitzende des ESMO Leadership‐Development Committees und erhielt Reisekostenzuschüsse von Neracare. Darüber hinaus berichtet sie über Honorare für die Durchführung klinischer Studien mit Zahlungen an die Institution von Agenus, AstraZeneca, Biontech, BMS, HUYA, iFIT, Immunocore, IO Biotech, MNI, MSD, Neracare, Novartis, Pascoe, Pfizer, Philogen, Regeneron, Roche, Sanofi, SkylineDx und Unicancer – außerhalb der eingereichten Arbeit. U.L. erhielt Zahlungen/Honorare für Vorträge von Sun Pharma und Sanofi sowie Reisekostenzuschüsse von Sun Pharma, Sanofi und Pierre Fabre. Sie ist zudem Boardmember bei Pierre Fabre, Sun Pharma, Sanofi, MSD, Novartis, DECOG, SCOPE und EADO – außerhalb der eingereichten Arbeit. L.F. erhielt Fördermittel von Hookipa Pharma, der Swiss Cancer League, der Deutschen Forschungsgemeinschaft, Immunophotonics und Mundipharma. Zudem erhielt sie Beratungshonorare von Philogen sowie Reisekostenzuschüsse von Philogen und Hookipa Pharma. Sie ist Mitglied des Vorstands der Universität Basel (TIL‐Studie, unbezahlte Tätigkeit) und Gründerin von Hookipa Pharma, Schmelzberg, Humion und Abtherix – außerhalb der eingereichten Arbeit. A.F. war als Beraterin für Novartis, MSD, BMS, Pierre Fabre und Immunocore tätig, erhielt Reisekostenzuschüsse von Novartis, BMS und Pierre Fabre sowie Honorare für Vorträge von Novartis, Delcath, BMS und MSD. Darüber hinaus erhielt sie institutionelle Forschungsstipendien von der BMS Stiftung Immunonkologie – außerhalb der eingereichten Arbeit. L.N., A.B. und N.S. erklären keine Interessenkonflikte.

## Supporting information



Supplementary information

Supplementary information
