# Supplementary material for: Psychosoziale Belastung und persistierende Nebenwirkungen bei Langzeitüberlebenden einer Melanomerkrankung im Stadium IV – eine Querschnittsbefragung
Source: J Dtsch Dermatol Ges. 2025 Jul 14;23(7):832–43. [Article in German] doi: 10.1111/ddg.15712_g (PMC12257057; doi:10.1111/ddg.15712_g)
Supplement: Supplementary file 1 — Supplementary information [file DDG-23-832-s001.docx]

| \| **DT** \| **N** \| \| **%** \| \| --- \| --- \| --- \| --- \| \| 0 \| 13 \| **56** \| 0 \| \| 1 \| 8 \| 1 \| \| 2 \| 18 \| 2 \| \| 3 \| 10 \| 3 \| \| 4 \| 7 \| **32** \| 4 \| \| 5 \| 13 \| 5 \| \| 6 \| 4 \| 6 \| \| 7 \| 10 \| 7 \| \| 8 \| 2 \| 8 \| \| 9 \| 2 \| 9 \| \| 10 \| 1 \| 10 \| \| **Total** \| **88** \| \| **100** \| | \| **HSI** \| **N** \| \| **%** \| \| --- \| --- \| --- \| --- \| \| 0 \| 26 \| **61** \| 28.9 \| \| 1 \| 5 \| 5.6 \| \| 2 \| 20 \| 22.2 \| \| 3 \| 10 \| 11.1 \| \| 4 \| 11 \| **29** \| 12.2 \| \| 5 \| 4 \| 4.4 \| \| 6 \| 5 \| 5.6 \| \| 7 \| 3 \| 3.3 \| \| 8 \| 3 \| 3.3 \| \| 9 \| 1 \| 1.1 \| \| 10 \| 2 \| 2.2 \| \| **Total** \| **90** \| \| **100** \| | \| **SA** \| **N** \| **%** \| \| --- \| --- \| --- \| \| Benötigt keine Hilfe \| **79** \| **87.8** \| \| Benötigt Hilfe \| **11** \| **12.2** \| \| **Total** \| **90** \| **100** \| |
| --- | --- | --- | --- | --- | --- | --- | --- | --- | --- | --- | --- | --- | --- | --- | --- | --- | --- | --- | --- | --- | --- | --- | --- | --- | --- | --- | --- | --- | --- | --- | --- | --- | --- | --- | --- | --- | --- | --- | --- | --- | --- | --- | --- | --- | --- | --- | --- | --- | --- | --- | --- | --- | --- | --- | --- | --- | --- | --- | --- | --- | --- | --- | --- | --- | --- | --- | --- | --- | --- | --- | --- | --- | --- | --- | --- | --- | --- | --- | --- | --- | --- | --- | --- | --- | --- | --- | --- | --- | --- | --- | --- | --- | --- | --- | --- | --- | --- | --- | --- | --- |

**Online Supplement Tabelle S2**

Online-Supplement Tabelle S2 gibt einen Überblick über die Ergebnisse der Selbsteinschätzung (SA), des Distress-Thermometers (DT) und des Hornheide-Screening-Instruments (HSI) bei Patientinnen und Patienten mit Melanomerkrankung im Stadium IV.
