# Supplementary material for: Psychosoziale Belastung und persistierende Nebenwirkungen bei Langzeitüberlebenden einer Melanomerkrankung im Stadium IV – eine Querschnittsbefragung
Source: J Dtsch Dermatol Ges. 2025 Jul 14;23(7):832–43. [Article in German] doi: 10.1111/ddg.15712_g (PMC12257057; doi:10.1111/ddg.15712_g)
Supplement: Supplementary file 2 — Supplementary information [file DDG-23-832-s002.docx]

| **Faktoren** | **Kate-gorie** | **N** | **B** | **SE** | **p** | **Odds ratio**  **[Exp(B)]** | **95 % KI Exp(B)** | | **Cox-/Snell R Quadrat** | **Nagelkerkes R-Quadrat** |
| --- | --- | --- | --- | --- | --- | --- | --- | --- | --- | --- |
|  |  |  |  |  |  |  | **Untere Grenze** | **Obere Grenze** |  |  |
| **Geschlecht** | MSQ | 88 | 0.666 | 1.947 | 0.141 | 1.947 | 0.802 | 4.726 | 0.024 | 0.034 |
| **Leidet immer noch unter Problemen aufgrund von operativen Verfahren** | MSQ | 80 | 0.579 | 0.562 | 0.303 | 1.785 | 0.593 | 5.371 | 0.142 | 0.193 |
| **Leidet immer noch unter Problemen aufgrund einer Strahlentherapie** | MSQ | 80 | 0.055 | 0.651 | 0.933 | 1.057 | 0.295 | 3.784 | 0.142 | 0.193 |
| **Unterstützung durch die psychoonkologische Beratung** | MSQ | 70 | 0.757 | 0.614 | 0.218 | 2.131 | 0.640 | 7.102 | 0.025 | 0.035 |
| **Unterstützung durch die Sozialberatung** | MSQ | 70 | 0.063 | 0.615 | 0.918 | 1.065 | 0.319 | 3.558 | 0.025 | 0.035 |
| **Bestes Ansprechen unter der 1. ST (CR, PR, SD vs. PD)** | MSQ | 75 | 0.405 | 0.527 | 0.442 | 1.500 | 0.534 | 4.214 | 0.008 | 0.011 |
| **Bestes Ansprechen unter der 2. ST (CR, PR, SD vs. PD)** | MSQ | 32 | -1.163 | 1.183 | 0.326 | 0.313 | 0.031 | 3.177 | 0.035 | 0.047 |
| **Noch immer unter einer Systemtherapie** | MSQ | 88 | -0.616 | 0.707 | 0.384 | 0.540 | 0.135 | 2.161 | 0.009 | 0.013 |
| **Nimmt regelmäßig an Vorsorgeprogrammen anderer Krebsentitäten teil (Darm-, Brust-, Prostatakrebs)** | MSQ | 85 | -1.466 | 0.798 | 0.066 | 0.231 | 0.048 | 1.102 | 0.050 | 0.069 |
| **Erlebt finanzielle Beeinträchtigungen aufgrund der Melanomerkrankung Stadium IV** | MSQ | 83 | 1.570 | 0.871 | 0.071 | 4.808 | 0.872 | 26.500 | 0.043 | 0.059 |
| **Wohnsituation** | NCCN | 78 | -20.261 | 40192.970 | 1.000 | 0.001 | 0.001 | - | 0.168 | 0.233 |
| **Versicherung** | NCCN | 78 | -21.845 | 25818.486 | 0.999 | 0.001 | 0.001 | - | 0.168 | 0.233 |
| **Arbeit/Schule** | NCCN | 78 | -20.261 | 40192.970 | 1.000 | 0.001 | 0.001 | - | 0.168 | 0.233 |
| **Kinderbetreuung** | NCCN | 78 | 22.144 | 40192.969 | 1.000 | 4141744977,929 | 0.001 | - | 0.168 | 0.233 |
| **Finanzielle Situation** | NCCN | 78 | 1.698 | 1.232 | 0.168 | 5.462 | 0.488 | 61.098 | 0.168 | 0.233 |
| **Umgang mit dem Partner/in** | NCCN | 84 | 0.365 | 0.803 | 0.650 | 1..440 | 0.299 | 0.6943 | 0.070 | 0.960 |
| **Umgang mit Kindern** | NCCN | 84 | -20,196 | 21546,627 | 0.999 | 0.001 | 0.001 | - | 0.070 | 0.960 |
| **Umgang mit Freunden** | NCCN | 84 | 41,122 | 33200,162 | 0.999 | 7.227E+17 | 0.001 | - | 0.070 | 0.960 |
| **Sorgen** | NCCN | 83 | -0.095 | 0.788 | 0.904 | 0.909 | 0.194 | 4.261 | 0.368 | 0.507 |
| **Traurigkeit** | NCCN | 83 | 2.393 | 0.870 | 0.263 | 2.550 | 0.495 | 13.133 | 0.368 | 0.507 |
| **Depression** | NCCN | 83 | 0.003 | 1.313 | 0.998 | 1.003 | 0.076 | 13.163 | 0.368 | 0.507 |
| **Verlust des Interesses an alltägl. Aktivitäten** | NCCN | 83 | 1.215 | 0.969 | 0.210 | 3.371 | 0.505 | 22.526 | 0.368 | 0.507 |
| **Belange in Bezug auf Gott** | NCCN | 79 | 21.608 | 40192,969 | 1.000 | 2423212264 | 0.001 | - | 0.027 | 0.037 |
| **Verlust des Glaubens** | NCCN | 79 | 0.247 | 0.946 | 0.794 | 1.280 | 0.201 | 8.168 | 0.027 | 0.037 |
| **Schmerzen** | NCCN | 73 | -3.052 | 1.866 | 0.102 | 0.047 | 0.001 | 1.831 | 0.522 | 0.733 |
| **Übelkeit** | NCCN | 73 | 25.922 | 14126.428 | 0.999 | 1.810E+11 | 0.000 | - | 0.522 | 0.733 |
| **Fatigue/** **Müdigkeit** | NCCN | 73 | -3.266 | 1.712 | 0.056 | 0.038 | 0.001 | 1.094 | 0.522 | 0.733 |
| **Waschen/Ankleiden** | NCCN | 73 | 2.694 | 2.378 | 0.257 | 14.795 | 0.140 | 1563.521 | 0.522 | 0.733 |
| **Äußeres Erscheinungsbild** | NCCN | 73 | 3.527 | 3.018 | 0.243 | 34.006 | 0.092 | 12607.828 | 0.522 | 0.733 |
| **Atmung** | NCCN | 73 | -1.088 | 2.108 | 0.606 | 0.337 | 0.005 | 20.979 | 0.522 | 0.733 |
| **Entzündungen im Mundbereich** | NCCN | 73 | 2.116 | 2.197 | 0.336 | 8.296 | 0.112 | 615.650 | 0.522 | 0.733 |
| **Trockener Mund** | NCCN | 73 | 2.483 | 1.907 | 0.193 | 11.981 | 0.285 | 503.183 | 0.522 | 0.733 |
| **Essen/Ernährung** | NCCN | 73 | -1.589 | 2.675 | 0.552 | 0.204 | 0.001 | 38.632 | 0.522 | 0.733 |
| **Verdauungsstörungen** | NCCN | 73 | 1.090 | 2.095 | 0.603 | 2.974 | 0.049 | 180.597 | 0.522 | 0.733 |
| **Obstipation** | NCCN | 73 | -1.919 | 2.384 | 0.421 | 0.174 | 0.001 | 15.697 | 0.522 | 0.733 |
| **Durchfall** | NCCN | 73 | 0.489 | 1.951 | 0.802 | 1.630 | 0.036 | 74.679 | 0.522 | 0.733 |
| **Probleme beim Wasserlassen** | NCCN | 73 | -0.892 | 2.470 | 0.718 | 0.410 | 0.003 | 51.935 | 0.522 | 0.733 |
| **Fieber** | NCCN | 73 | 1.568 | 3.118 | 0.615 | 4.799 | 0,011 | 2165.587 | 0.522 | 0.733 |
| **Trockene/verstopfte Nase** | NCCN | 73 | 0.735 | 1.464 | 0.616 | 2.085 | 0.118 | 36.755 | 0.522 | 0.733 |
| **Kribbeln in Händen/Füßen** | NCCN | 73 | -3.854 | 2.135 | 0.071 | 0.021 | 0.001 | 1.391 | 0.522 | 0.733 |
| **Angeschwollen/**  **aufgedunsen fühlen** | NCCN | 73 | 1.254 | 1.817 | 0.490 | 3.505 | 0.099 | 123.512 | 0.522 | 0.733 |
| **Gedächtnis/**  **Konzentration** | NCCN | 73 | -0.108 | 1.511 | 0.943 | 0.898 | 0.046 | 17.362 | 0.522 | 0.733 |
| **Sexuelle Probleme** | NCCN | 73 | -6.559 | 3.838 | 0.087 | 0.001 | 0.001 | 2.621 | 0.522 | 0.733 |

**Online Supplement Tabelle S3 Faktoren, die nicht signifikant mit einer erhöhten Belastung im DT (Cut-off ≥5) assoziiert sind**

Eine binäre logistische Regression wurde für die NCCN-Problem-Liste und die MSQ (melanomspezifischen Fragen) durchgeführt, um Faktoren zu identifizieren, die signifikant mit einer erhöhten Belastung im DT bzw. dem Bedarf an psychoonkologischer Unterstützung (Cut-off ≥5) assoziiert sind. Gibt das Modell keine obere Grenze für das KI (Konfidenzintervall) an, ist diese entweder undefiniert oder unendlich. Das Fehlen einer oberen Grenze weist auf eine hohe Unsicherheit des Modells bei der Schätzung der Odds Ratio hin, was die Interpretation und Verlässlichkeit des Faktors erheblich einschränkt. Die folgenden Abkürzungen werden verwendet: NCCN (National Comprehensive Cancer Network Problem-Liste), MSQ (melanomspezifische Fragen), KI (Konfidenzintervall), Exp(B) (Exponentialfunktion von B). (Signifikanz), SE (Standardfehler).
